# Assessment of endotracheal aspirate culture appropriateness among adult ICU patients at an academic medical center

**DOI:** 10.1017/ash.2023.308

**Published:** 2023-09-29

**Authors:** Michael Chambers, Romney Humphries, Bryan Harris, Tom Talbot

## Abstract

**Background:** Ventilator-associated pneumonia (VAP) is a significant cause of mortality in intensive care units (ICUs), but minimal research exists regarding the appropriateness of ordering endotracheal aspirate cultures (EACs). We evaluated the diagnostic utility of rationales given for EAC collection in ICUs at an academic medical center to assess potentially inappropriate EAC ordering. **Methods:** The study population comprised all adult patients admitted to an ICU in 2019 who underwent EAC collection. A random 10% sample from this population, stratified by ICU type, was selected. Clinical and diagnostic characteristics within 24 hours of EAC collection were identified by chart review. Clinical documentation was reviewed to identify ICU provider rationales for ordering EAC. **Results:** In total, 749 patients underwent EAC collection. Among them, 75 patients comprised the random sample, of whom 7 (9.3%) were excluded due to extubation before culture collection. Figure 1 shows patient distribution by ICU type. From these 68 patients, 105 EACs were collected. Of these, 41 (39%) were positive for potential pathogens, and 59 (56.2%) had explicit rationales for EAC collection, including fever (44.1%), hypoxia (18.6%), leukocytosis (16.9%), secretions (11.9%), shock (10.2%), and radiologic findings (8.5%). Also, 43.8% of EACs had no explicit rationale for collection. Table 1 shows sensitivities, specificities, positive likelihood ratios (LRs), and negative LRs for these rationales and related characteristics. **Conclusions:** EACs were commonly ordered without clear clinical indications. Of the noted rationales for EAC collections, most performed poorly at predicting positive cultures, which challenged common rationales for ordering EAC. This study could serve as a foundation for diagnostic stewardship interventions for EAC, potentially decreasing unnecessary cultures.

**Figure:**
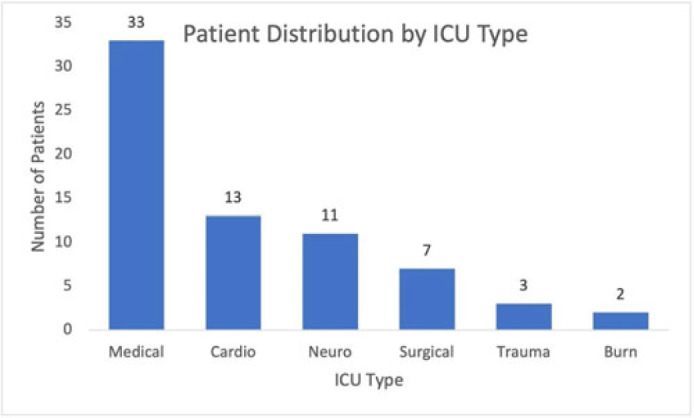

**Figure:**
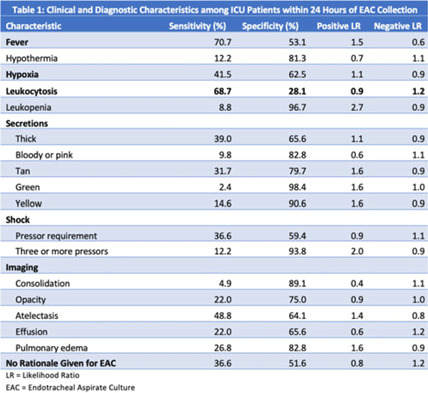

**Disclosures:** None

